# Early detection of myocardial involvement by non-contrast T1ρ mapping of cardiac magnetic resonance in type 2 diabetes mellitus

**DOI:** 10.3389/fendo.2024.1335899

**Published:** 2024-03-05

**Authors:** Hongmin Shu, Huimin Xu, Zixiang Pan, Yan Liu, Wei Deng, Ren Zhao, Yan Sun, Zhen Wang, Jinxiu Yang, Hui Gao, Kaixuan Yao, Jie Zheng, Yongqiang Yu, Xiaohu Li

**Affiliations:** ^1^ Department of Radiology, the First Affiliated Hospital of Anhui Medical University, Research Center of Clinical Medical Imaging, Anhui Province Clinical Image Quality Control Center, Hefei, Anhui, China; ^2^ Department of Cardiology, the First Affiliated Hospital of Anhui Medical University, Hefei, Anhui, China; ^3^ Department of Geriatric Endocrinology, the First Affiliated Hospital of Anhui Medical University, Hefei, Anhui, China; ^4^ Mallinckrodt Institute of Radiology, Washington University School of Medicine in St. Louis, St. Louis, MO, United States

**Keywords:** diabetes mellitus, myocardial diffuse fibrosis, T1ρ mapping, spin-lock, extracellular volume fraction

## Abstract

**Objective:**

This study aims to determine the effectiveness of T1ρ in detecting myocardial fibrosis in type 2 diabetes mellitus (T2DM) patients by comparing with native T1 and extracellular volume (ECV) fraction.

**Methods:**

T2DM patients (n = 35) and healthy controls (n = 30) underwent cardiac magnetic resonance. ECV, T1ρ, native T1, and global longitudinal strain (GLS) values were assessed. Diagnostic performance was analyzed using receiver operating curves.

**Results:**

The global ECV and T1ρ of T2DM group (ECV = 32.1 ± 3.2%, T1ρ = 51.6 ± 3.8 msec) were significantly higher than those of controls (ECV = 26.2 ± 1.6%, T1ρ = 46.8 ± 2.0 msec) (all P < 0.001), whether there was no significant difference in native T1 between T2DM and controls (P = 0.264). The GLS decreased significantly in T2DM patients compared with controls (−16.5 ± 2.4% vs. −18.3 ± 2.6%, P = 0.015). The T1ρ and native T1 were associated with ECV (Pearson’s r = 0.50 and 0.25, respectively, both P < 0.001); the native T1, T1ρ, and ECV were associated with hemoglobin A1c (Pearson’s r = 0.41, 0.52, and 0.61, respectively, all P < 0.05); and the ECV was associated with diabetes duration (Pearson’s r = 0.41, P = 0.016). The AUC of ECV, T1ρ, GLS, and native T1 were 0.869, 0.810, 0.659, and 0.524, respectively.

**Conclusion:**

In T2DM patients, T1ρ may be a new non-contrast cardiac magnetic resonance technique for identifying myocardial diffuse fibrosis, and T1ρ may be more sensitive than native T1 in the detection of myocardial diffuse fibrosis.

## Introduction

For individuals with diabetes, the most common cause of illness and death is cardiovascular disease (1). This condition often triggers cardiac relaxation irregularities, primarily causing heart failure with preserved ejection fraction (2). A variety of molecular processes contribute to the death of heart muscle cells and widespread fibrosis in the heart muscle, culminating in impaired heart function and heart failure (3). Previous studies (4, 5) have shown that diabetes-associated myocardial fibrosis is mainly caused by factors such as hyperglycemia, lipotoxicity, and insulin resistance. These factors stimulate cardiac fibroblasts, leading to increased matrix secretion and paracrine signaling activation in cardiomyocytes, immune cells, and vascular cells, which further promotes fibroblast activation. In addition, the progression of diabetic fibrosis is associated with various other factors, including neurohormonal pathways, cytokines, growth factors, oxidative stress, advanced glycation end products (AGEs), and matricellular proteins. These factors lead to complex interactions that ultimately result in myocardial fibrosis in T2DM patients. Therefore, early identification of myocardial diffuse fibrosis might be important to improve outcomes.

Myocardial native T1 is suggested for the assessment of diffuse fibrosis, but it has limited sensitivity and is scanner dependent (6). The T1 mapping-derived extracellular volume (ECV) fraction is currently the reference imaging marker for the assessment of myocardial diffuse fibrosis (7). Compared with the native T1, ECV showed higher repeatability and comparability in the diagnosis of myocardial diffuse fibrosis. However, the ECV value needs to be calculated based on the T1 mapping value before and after contrast medium injection, which is limited in patients with gadolinium contraindications.

T1ρ, or the spin-lattice relaxation time in a rotating frame, emerges as a novel quantitative metric for potentially evaluating myocardial diffuse fibrosis (8). The T1ρ value describes the relaxation time after flipping the magnetization and locking it by a spin-locking (SL) radio frequency (RF) pulse (9). It was well documented that T1ρ is sensitive to the slow motion of the water proton affiliated with large macromolecules (10). T1ρ, through specific RF pulse adjustments, can provide insights into myocardial tissue macromolecules like collagen (9). Studies have demonstrated the capability of T1ρ mapping to identify early stages of myocardial diffuse fibrosis in diabetic monkeys (11). However, T1ρ has not been reported in patients with type 2 diabetes mellitus (T2DM). In this project, the purpose of this study is to evaluate the feasibility of T1ρ in detecting myocardial diffuse fibrosis in T2DM in a comparison with myocardial native T1 and ECV.

## Materials and methods

### Study population

A total of 35 patients with T2DM (age 52 ± 12 years, 60% men) were recruited prospectively in the First Affiliated Hospital of Anhui Medical University from March 2022 to November 2022. To be included, T2DM patients had to be diagnosed according to World Health Organization guidelines (12) and free from any history of cardiac issues, including coronary artery disease, congenital heart disorders, cardiomyopathy, or valvular heart disease. Additionally, they should not exhibit symptoms like chest pain, palpitations, or shortness of breath and must have normal electrocardiogram results. Participants were excluded if they had renal dysfunction, contraindications for undergoing MRI, diabetes with uncontrolled hypertension, or left ventricular hypertrophy, defined as a cardiac MRI-derived left ventricular myocardial mass exceeding 61 g/m² in women or 81 g/m² in men when indexed to body surface area, following the criteria set by Olivotto et al. (13). Patients’ clinical data, family history, and echocardiographic results were acquired from the electronic medical records. In addition, we recruited 30 healthy controls (age 49 ± 7 years, 67% men) matched by age, sex, and BMI, and without cardiovascular disease risk factors, family cardiac history, previous hospital admissions, or cardiovascular medications. The characteristics of subjects are listed in **Table 1**. The Ethics Committee of the First Affiliated Hospital of Anhui Medical University approved this study, and all participants provided written consent before involvement (approval number: PJ2022-09-60).

**Table 1:** Baseline characteristics of the patients and healthy controls.

Parameters	Controls(N = 30)	T2DM (N = 35)	*P*
Age (years)	49 ± 7	52 ± 12	0.164
Gender			0.579
Men	20 (67%)	21 (60%)	–
Women	10 (33%)	14 (40%)	–
Disease duration (years)	–	7.2 ± 5.4	–
BSA (m^2^)	1.7 ± 0.2	1.7 ± 0.2	0.716
BMI (kg/m^2^)	23.1 ± 4.1	24.7 ± 3.1	0.077
HR (beats/min)	70.5 ± 6.7	72.6 ± 10.8	0.342
Systolic blood pressure (mmHg)	126.6 ± 9.1	129.3 ± 10.2	0.134
Diastolic blood pressure (mmHg)	77.6 ± 9.8	80.5 ± 6.7	0.156
History of smoking	0(%)	5 (14%)	0.057
Blood glucose level (mmol/L)	–	10.9 ± 5.2	–
Hemoglobin (g/L)	–	129.2 ± 22.5	–
Hemoglobin A1C (%)	–	8.0 ± 1.5	–
Creatinine (μmol/L)	–	66.7 ± 15.2	–
Total cholesterol (mmol/L)	–	4.6 ± 1.1	–
Triglycerides (mmol/L)	–	2.0 ± 1.2	–
HDL-C (mmol/L)	–	1.1 ± 0.3	–
LDL-C (mmol/L)	–	2.8 ± 0.9	–

Continuous variables are presented as mean ± standard deviation, and categorical variables are presented as N (%). BSA, body surface area; BMI, body mass index; HR, heart rate; HDL-C, high-density lipoprotein cholesterol; LDL-C, low-density lipoprotein cholesterol.

### Cardiac MRI protocol

Cardiac MRI was conducted using a 1.5-T whole-body MRI system (Ingenia, Philips Healthcare, Best, Netherlands). Patients were scanned using a 32-element body array coil. Cardiac cine magnetic resonance imaging was performed using a balanced steady-state free precession sequence with breath-holding. Contiguous parallel short-axis views covering the full left ventricle (LV) and right ventricle (RV) from base to apex were acquired, along with standard long-axis two-, three-, and four-chamber views. Native T1 mapping and postcontrast T1 mapping were performed at the basal, middle, and apical levels of the LV short-axis using a Modified Look-Locker Inversion recovery (MOLLI, 5(3)3 protocol) and MOLLI (4(1)3(1)2) protocol) sequence, respectively. T2 mapping was performed using a multiecho gradient spin echo (GraSE) sequence, with the slice location kept the same as for T1 mapping. Late gadolinium enhancement imaging was performed using a segmented phase-sensitive inversion recovery sequence within 10 to 15 min after injection of a gadolinium contrast agent, comprising the same views as cine images. T1ρ mapping was performed at the same basal, middle, and apical levels of the LV short axis as the T1 mapping slices using an SL-prepared steady-state free precession sequence. Each SL pulse consisted of two continuous RF pulses with opposite phases to compensate for B1 variations, and a refocusing pulse between the spin-locking halves to compensate for B0 errors. Images acquired with four different times of spin locking (TSL) (0, 10, 20, 30 ms) were used to calculate each T1ρ map. These four images were acquired within a single breath-hold scan triggered by an electrocardiogram signal. Each T1ρ-weighted image was acquired at mid-diastole to minimize cardiac motion, with an acquisition window of approximately 250 ms. All the cardiac MRI parameters are summarized in **Table 2**.

**Table 2:** Cardiac MRI parameters.

Parameters	Cine	T1 mapping	T2 mapping	LGE	T1_ρ_ mapping
FOV (mm^2^)	300 × 300	300 × 300	300 × 300	300 × 300	271 × 271
Slice thickness (mm)	8	7	10	6	10
Acquired pixel size (mm^2^)	1.97 × 2.26	2.0 × 2.0	2.0 × 2.0	1.6 × 1.96	3.0 × 3.0
Recon voxel size (mm^2^)	1.04 × 1.04	1.17 × 1.17	1.04 × 1.04	0.89 × 0.89	0.85 × 0.85
Matrix size	152 × 133	152 × 150	152 × 148	188 × 153	64 × 64
SENSE	2	2	3	2	2
TR/TE (ms)	3.4/1.52	3.0/1.40	750/9.3(ΔTE)	6.0/3.0	2.5/1.24
Spin-locking frequency (Hz)	None	None	None	None	400
TSL (ms)	None	None	None	None	0, 10, 20, 30
Flip angle (°)	60	35	90	25	60

FOV, field of view; TSL, time of spin locking.

### Postprocessing and analysis

Cvi42 (Version 5.6.6, Circle Cardiovascular Imaging Inc., Calgary, Canada) was utilized for the automatic delineation of endocardial and epicardial borders (with manual adjustments when needed) at the end of the systolic and diastolic phases of the stacks of short-axis cine images, and cardiac functional parameters were automatically calculated and generated. Native T1, T2, and postcontrast T1 maps were produced by importing respective images into their corresponding analysis modules. T1ρ maps were calculated by pixelwise fitting of a monoexponential decay function using a Levenberg–Marquardt algorithm of non-linear estimation (14) using Matlab (Version R2018b; MathWorks, Natick, MA). For global measurements of native T1, T2, and T1ρ values, the software automatically generated these values once the contours on the basal, middle, and apical slices were delineated. Venous blood samples, collected within 24 h prior to the MRI scans, were analyzed for hematocrit levels. ECV, derived from cardiac MRI, was then calculated as previously described (15). Cardiac MRI feature tracking was performed using Cvi42, with two-dimensional global peak LV strain from feature tracking evaluated as per earlier studies (16). Native T1 maps, T2 maps,T1ρ maps, and ECV maps were analyzed using the 16 AHA (17) segments of the LV (excluding the apical segment) by two radiologists (H.S. and H.X., each with 2 years of experience in cardiac MRI evaluation) blinded to patient information. Any differences between the two observers were adjudicated by a senior observer (X.L., with 15 years’ experience in MRI). At our center, the upper normal limits for native T1, T2, ECV, and T1ρ were set at 1,090 ms, 57.0 ms, 29%, and 51 ms, respectively. Prolonged values of native T1, T1ρ, and ECV in any of the 16 AHA segments indicated myocardial involvement.

The reliability of intraobserver measurements was evaluated by repeating assessments on 15 randomly chosen subjects by one radiologist, blind to initial results, at least a week apart. Another radiologist, unaware of the first’s measurements, independently assessed interobserver reliability on 15 subjects.

### Statistical analysis

Statistical analysis was performed using SPSS (version 26.0, Statistical Package for the Social Sciences, International Business Machines, Inc, Armonk, New York, USA) and GraphPad Prism (version 9.0 GraphPad Inc. USA). A P-value less than 0.05 was considered indicative of statistical significance. The Shapiro–Wilk test was applied to determine the normality of data distribution. Continuous variables that followed a normal distribution (as determined by the Kolmogorov–Smirnov test with P ≥ 0.05) were presented as mean ± standard deviation. The Student’s t-test was employed to compare differences in continuous variables. Categorical data were represented as N (%) and analyzed using Fisher’s exact test for differences. Linear relationships between variables were investigated using the Pearson correlation coefficient (r). The ability of ECV, T1ρ GLS, and native T1 to differentiate between T2DM patients and control subjects was assessed through receiver operating characteristic (ROC) curve analysis. The AUCs were compared using the Delong test.

## Results

### Patient characteristics

**Figure 1:**
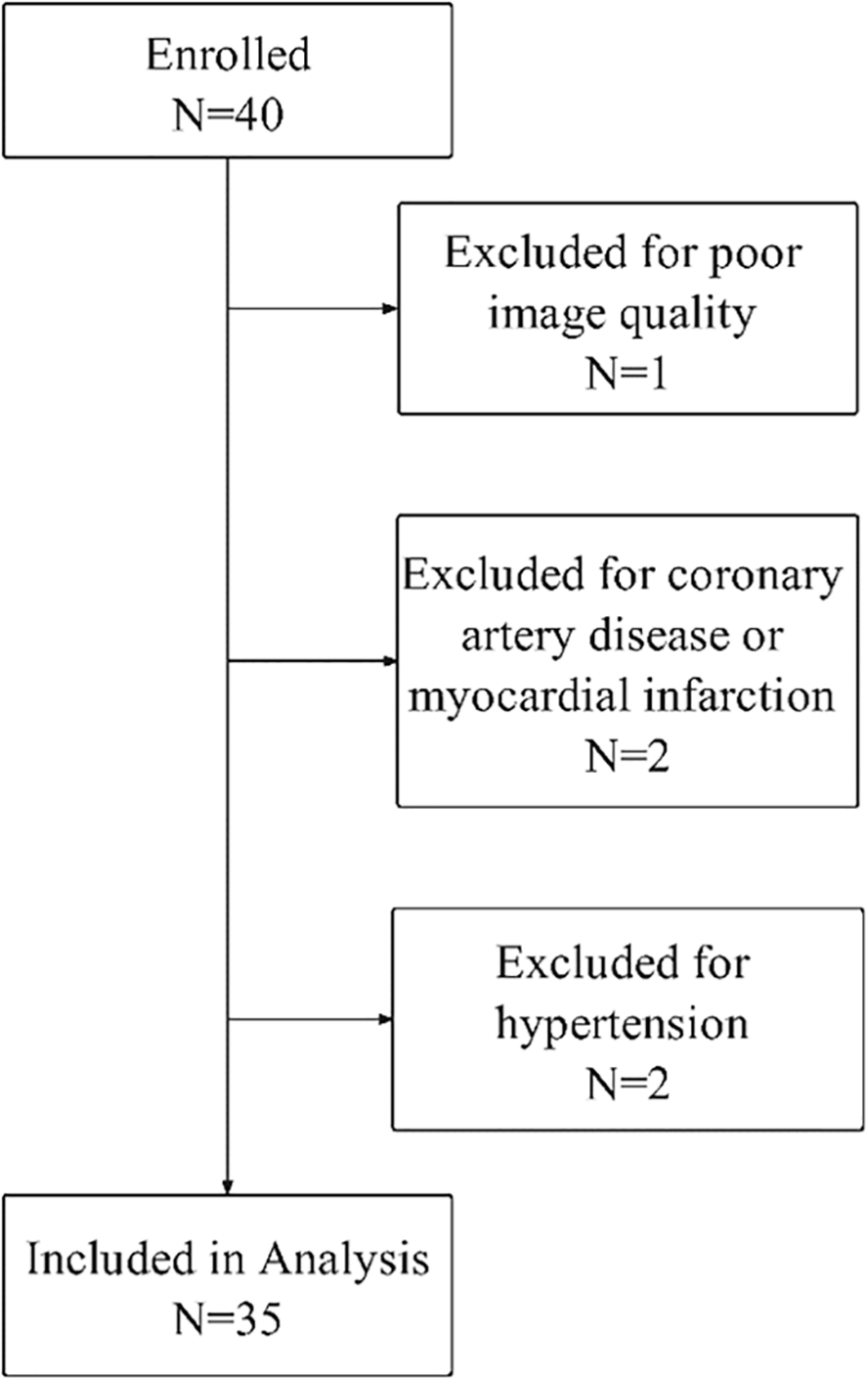
Flowchart of study participants. A total of 40 patients were enrolled. Patients were excluded for poor image quality (N = 1), coronary artery disease or myocardial infarction (N = 2), and hypertension (N = 2). The final T2DM patient group included 35 patients.

A total of 40 patients were enrolled, and 5 patients were excluded for poor image quality (N = 1), coronary artery disease or myocardial infarction (N = 2), and hypertension (N = 2). The final non-ischemic cardiomyopathy group included 35 patients (age 52 ± 12 years, 60% men) and 30 healthy controls (age 49 ± 7 years, 67% men) (**Figure 1**). Doppler ultrasound showed diastolic dysfunction in five patients (the ratio between E-wave and A-wave < 1). The baseline characteristics of the study population are presented in **Table 1**.

### Cardiac MRI measurements

**Figure 2:**
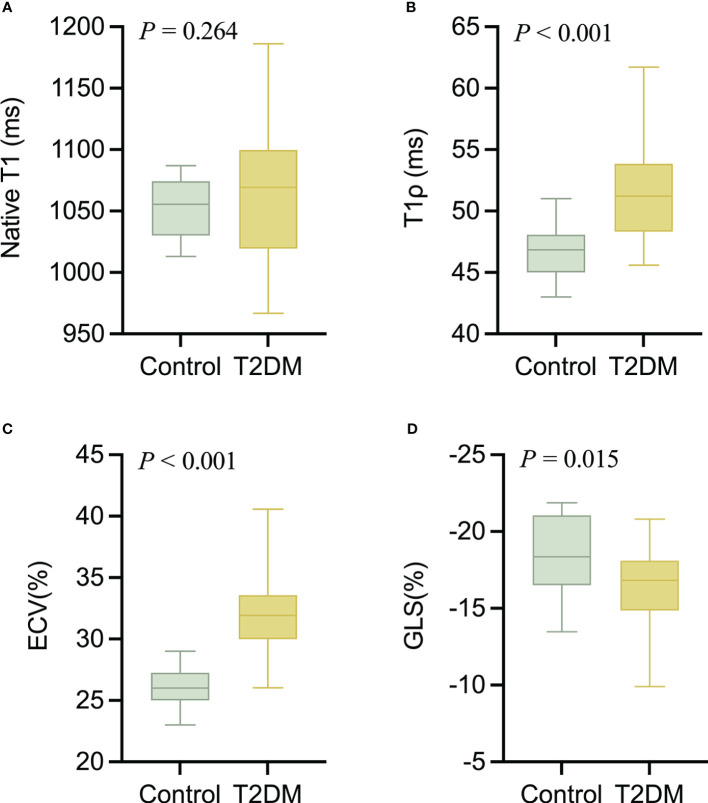
The comparison of nativeT1 **(A)**, T1ρ **(B)**, ECV **(C)**, and GLS **(D)** values between T2DM group and controls.

**Figure 3:**
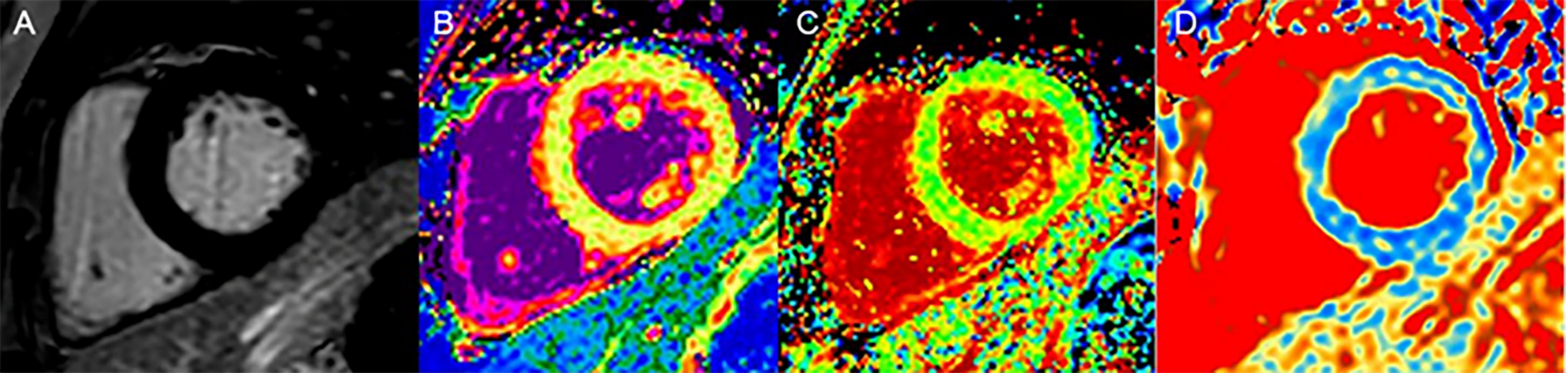
A 60-year-old woman with DM, LGE is negative **(A)**; native T1 values (1,056 ms), no statistical difference compared with the controls **(B)**; ECV values (31%) were elevated **(C)**; T1ρ values (52 ms) were elevated **(D)**.

**Figure 4:**
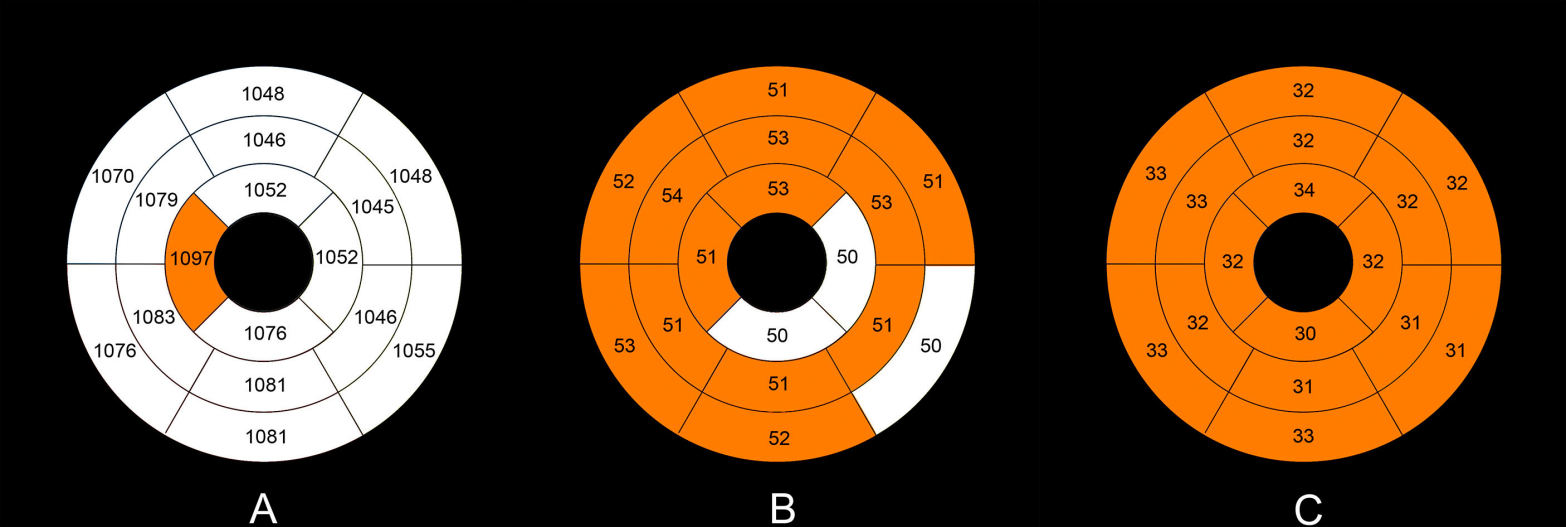
Mean native T1 **(A)**, T1ρ **(B)**, and ECV values **(C)** in the T2DM group in an AHA 16-segment model display. The native T1, T1ρ, and ECV values of more than the upper limits (mean + 2×standard deviation) of the normal values (native T1 1,090 ms, T1ρ 51 ms, ECV 29%) are shown with orange color.

The intra- and interobserver variabilities of native T1 and T1ρ are similar (**Table 3**). Cardiac MRI measurements for all subjects are shown in **Table 4**. The global ECV and T1ρ values of the T2DM group (ECV = 32.1 ± 3.2%, T1ρ = 51.6 ± 3.8 msec) were significantly higher than those of controls (ECV = 26.2 ± 1.6%, T1ρ = 46.8 ± 2.0 msec) (all *P* < 0.001) (**Figures 2**, **3**). Compared with the controls, the global longitudinal strain (GLS) decreased significantly in T2DM patients (−16.5 ± 2.4% vs. −18.3 ± 2.6%, *P* = 0.015). There was no significant difference in global T2 values, left ventricular ejection fraction (LVEF), left ventricular end-diastolic volume index (LVEDVi), left ventricular end-systolic volume index (LVESVi), left ventricular stroke volume index (LVSVi), LV mass index, and native T1 between T2DM and controls (all *P* > 0.05). The 16 AHA segments of global native T1, T1ρ, and ECV in patients with T2DM were showed in the bullseye display (**Figure 4**).

**Table 3:** Intra- and interobserver variability of native T1 and T1ρ.

Parameters	Intraobserver		Interobserver	
	ICC	95% CI	ICC	95% CI
Native T1 (ms)	0.95	0.93~0.98	0.94	0.87~0.98
T1ρ (ms)	0.96	0.92~0.98	0.89	0.71~0.94

ICC, intra-class correlation coefficient; CI, confidence interval.

**Table 4:** Cardiac MRI findings.

Parameters	Controls (N = 30)	T2DM (N = 35)	*P*
Left ventricle (LV)			
LVEF (%)	60.3 ± 6.1	58.2 ± 6.9	0.209
LVEDVi (mL/m^2^)	78.6 ± 9.3	73.4 ± 16.0	0.109
LVESVi (mL/m^2^)	31.6 ± 6.4	30.4 ± 8.4	0.532
LVSVi (mL/m^2^)	47.0 ± 7.4	43.0 ± 10.3	0.079
LV mass index (g/m^2^)	46.0 ± 9.0	51.4 ± 13.6	0.072
Tissue characterization			
Global T1 (ms)	1,053.0 ± 23.4	1,064.8 ± 55.9	0.264
Global T2 (ms)	53.2 ± 1.9	54.0 ± 2.1	0.108
Global ECV (%)	26.2 ± 1.6	32.1 ± 3.2	< 0.001*
Global T1ρ (ms)	46.8 ± 2.0	51.6 ± 3.8	< 0.001*
Global GLS (%)	−18.3 ± 2.6	−16.5 ± 2.4	0.015*

Values are presented as mean ± SD. Differences between groups were calculated using Student’s t-test. LVEF, left ventricular ejection fraction; LVEDVi, left ventricular end-diastolic volume index; LVESVi, left ventricular end-systolic volume index; LVSVi, left ventricular stroke volume index; ECV, extracellular volume. *indicates a statistically significant difference.

### Correlation analysis

**Figure 5:**
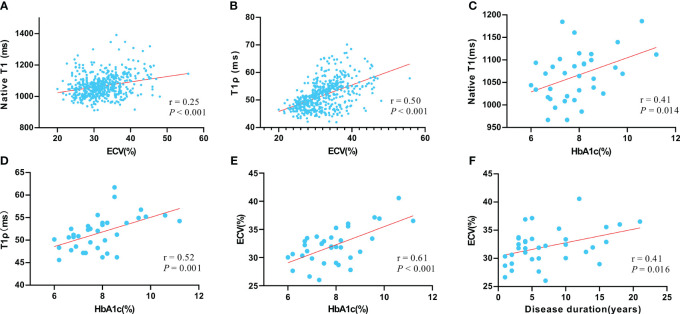
The correlation between native T1 value and ECV value **(A)**; the correlation between T1ρ value and ECV value **(B)**; the correlation between native T1 value and hemoglobin A1c (HbA1c) **(C)**; the correlation between T1ρ value and HbA1c **(D)**; the correlation between native ECV value and HbA1c **(E)**; the correlation between ECV and disease duration **(F)**. Pearson correlation was used to assess linear relationships between variables.

Correlation analysis was performed in all T2DM patients. The T1ρ and native T1 values were significantly correlated with ECV (Pearson’s r = 0.50 and 0.25, respectively, both *P* < 0.001) on the segment basis. The global native T1, T1ρ, and ECV were moderately or strongly correlated with hemoglobin A1c (Pearson’s r = 0.41 0.52, and 0.61, respectively, all *P* < 0.05) on a patient basis. The global ECV was also moderately correlated with diabetes duration (Pearson’s r = 0.41, *P* = 0.016) (**Figure 5**).

### ROC curve analysis for diagnosis of T2DM patients and controls

**Figure 6:**
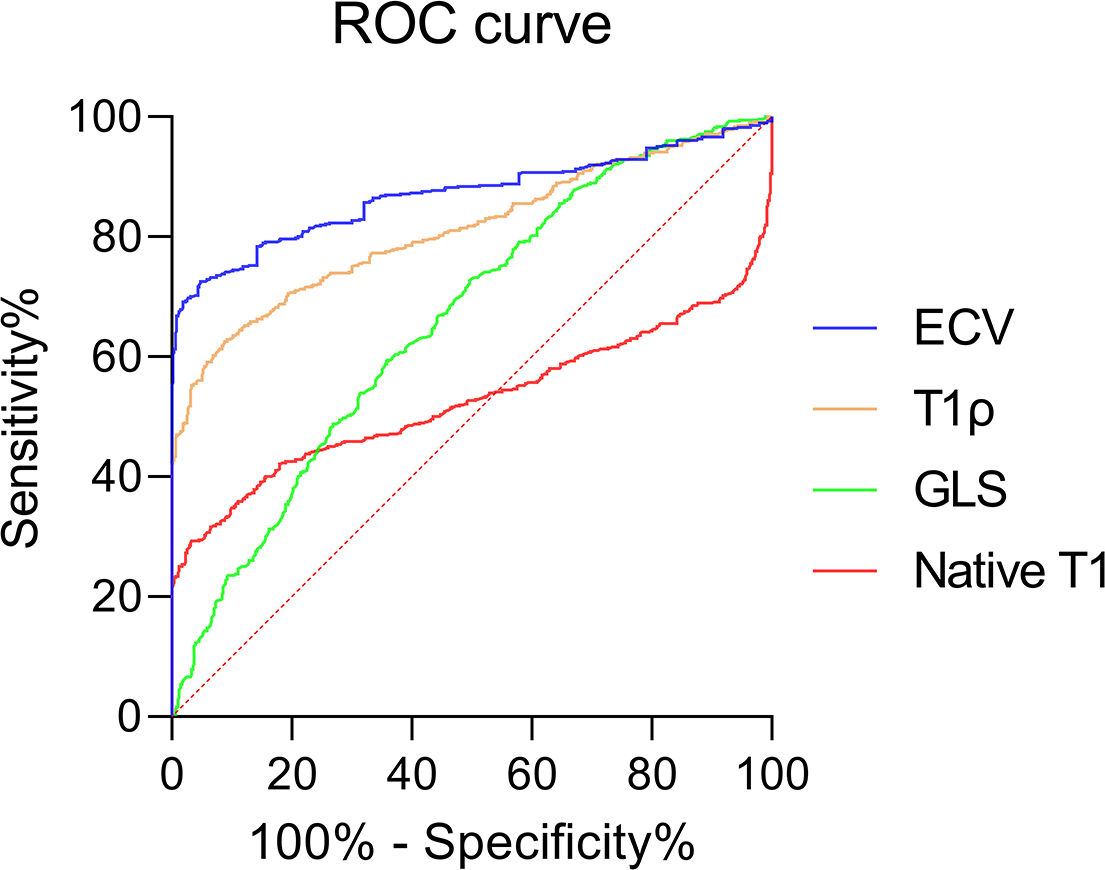
Receiver operating curves showing the diagnostic performance of ECV, T1ρ GLS, and native T1 in distinguishing T2DM patients and controls.

The AUCs of ECV, T1ρ, GLS, and native T1 were 0.869, 0.810, 0.659, and 0.524, and there was a statistical difference between any two of them (all *P* < 0.001) (**Figure 6**). The best cutoff values of ECV, T1ρ, GLS, and native T1 were 28.9% (sensitivity 72.5%, specificity 95.2%), 49.5 ms (sensitivity 62.5%, specificity 91.2%), −17.2% (sensitivity 59.3%, specificity 64.2%), and 1,094.2 ms (sensitivity 29.1%, specificity 96.7%).

## Discussion

As far as we are aware, this is the first report of the use of a non-contrast T1ρ technique to detect myocardial diffuse fibrosis in T2DM patients. Our findings indicate that T1ρ and ECV values in T2DM patients are notably higher compared with healthy individuals, although no significant differences in the left ventricular systolic dysfunction and native T1 value were observed between the two groups. In addition, the GLS was significantly lower in T2DM. The non-contrast T1ρ were significantly correlated with ECV and hemoglobin A1c. This compiling evidence suggests that T1ρ may be an alternative non-contrast method for non-invasive diagnosis of myocardial diffuse fibrosis in early T2DM.

The T1ρ relaxation time reflects the decay of longitudinal magnetization in a spin-lock radiofrequency field, capable of characterizing the ^1^H exchange between protein and water, and is particularly sensitive to low-frequency processes, showing promise in myocardial fibrosis detection. In this study, T1ρ showed an excellent ability to distinguish T2DM patients from controls. Zhang et al. (11) performed T1ρ mapping and histological analysis in T2DM monkeys. The results showed that T1ρ values increased significantly compared with controls, which demonstrated that the T1ρ method is a feasible non-contrast cardiac MRI technique for detecting myocardial fibrosis. In addition, previous studies (6, 15, 18–21) had also found that the myocardial T1ρ values in hypertrophic cardiomyopathy, dilated cardiomyopathy, end-stage renal disease, and myocardial infarction were significantly higher than those in the controls. Our results suggested that the cardiac MRI T1ρ technique can detect subclinical myocardial damage before patients developed overt cardiac dysfunction.

ECV is currently a reference biomarker of myocardial diffuse fibrosis (7). The finding of ECV in our study was consistent with others in which ECV values of patients with T2DM were significantly higher than those in healthy controls (22). However, Levelt et al. (23) reported that no significant difference in ECV was observed between T2DM and the control group. This may be partially attributed by different inclusion criteria. In Levelt’s study, T2DM patients with HBA1C >9% or those on insulin therapy were excluded. These patients were included in those studies aforementioned, including ours. There was a moderate correlation between ECV and T1ρ and a mild correlation between ECV and native T1 in our study. The same findings were also reported in a previous study (18). The correlation between ECV and T1ρ suggests that the expansion of myocardial interstitial might be related to myocardial collagen deposition, but this needed to be verified by pathology. ECV was acquired by native T1 value and postcontrast T1 value, which might explain the correlation between native T1 values and ECV.

Native T1 mapping in cardiac MRI is viewed as a non-contrast method for quantifying myocardial diffuse fibrosis (24). However, no significant difference in native T1 value was observed between the two groups. Liu et al. (22) found that there was no significant difference in native T1 between short-term (≤5 years) T2DM patients or longer-term (>5 years) T2DM patients and the controls, but the significant difference was observed in ECV. Based on these data, the sensitivity of native T1 in detecting myocardial diffuse fibrosis in T2DM may still be inferior to ECV and T1ρ.

GLS is considered to be a better cardiac MRI technology for assessing myocardial subclinical damage (25). In our study, the T2DM group exhibited a significant decrease in GLS, around 2%, compared with controls. The result was consistent with the findings of the study by Xie et al. (26). Xie found that most strain abnormalities in T2DM patients occurred in the GLS and were associated with myocardial diastolic function. Moreover, previous studies had shown that most global strain abnormalities first appear longitudinally, suggesting that longitudinal strain injury may occur early in the disease (27, 28).

In our study, there were five patients with diastolic dysfunction reported by Doppler ultrasound, with the lower LVEF (47.2 ± 2.0%) and GLS (−14.4 ± 2.6%), higher ECV (34.1 ± 2.6%), and T1ρ values (53.9 ± 5.6 ms), compared with T2DM patients with preserved LVEF. In a study of diabetic monkeys, monkeys with moderate diastolic dysfunction had lower GLS and higher ECV and T1ρ values, compared with monkeys with mild diastolic dysfunction (11). Dusenbery et al. (29) also demonstrated that elevated ECV correlated with echocardiographic indicators of diastolic dysfunction. Therefore, our findings suggested that although LVEF in most T2DM patients was normal, early myocardial diastolic dysfunction might be present.

The significant relationship between hemoglobin A1c levels and native T1, T1ρ, or ECV implies that myocardial interstitial expansion could be linked to inadequate glycemic control. A previous study (30) indicated a substantial correlation between both T1 and ECV with hemoglobin A1c levels in T2DM patients. Moreover, it was reported that hemoglobin A1c levels negatively correlated with systolic strain and diastolic function in T2DM patients (31). These insights suggest that chronic high blood sugar negatively impacts myocardial interstitial matrix expansion and early cardiac dysfunction. ECV could be used to quantify the extracellular volume fraction to characterize the degree of myocardial interstitial dilatation, which was related to myocardial diastolic dysfunction. Therefore, this could explain the correlation between ECV and hemoglobin A1c level. Previous studies had shown that T1ρ has a good correlation with ECV in detecting diffuse myocardial fibrosis, which was related to myocardial diastolic dysfunction (18, 32). This might be a possible explanation for the correlation between T1ρ and hemoglobin A1c. In addition, ECV was significantly correlated with disease duration in our study, which was consistent with the findings of Shang et al. (33). Previous studies have indicated that hyperglycemia, even transient spikes, can cause continuous cellular damage by disrupting signal feedback circuits, and promote cardiac fibroblast proliferation (34). The link between disease duration and ECV suggests that ECV might reflect the long-term impact of T2DM on the myocardium, a hypothesis that requires verification through further research with more participants.

Our research has several limitations. First, the sample size of this study is relatively small and further studies with more patients are needed to clarify the diagnostic value of T1ρ in T2DM with different comorbidities. Second, there was no histological validation for the presence of myocardial diffuse fibrosis. However, given several validation studies in ECV (35, 36), the close correlation of T1 p and ECV supports our conclusion. Third, within the healthy control group, there is the possibility of unrecognized prediabetes mellitus. This may underestimate the differences between the healthy controls and T2DM groups. Fourth, due to the limitations of single-center studies, caution should be exercised in interpretation. The lack of multicenter data limits the generalizability of our results. Future studies involving multiple centers will enhance the reliability and applicability of our findings to a broader population.

## Conclusion

In conclusion, this study demonstrates that an increased myocardial T1ρ can be detected prior to LV systolic dysfunction in patients with T2DM. Given the superior sensitivity of T1ρ over native T1, T1ρ might be a better non-contrast biomarker for assessing myocardial diffuse fibrosis in T2DM.

## Data availability statement

The original contributions presented in the study are included in the article. Further inquiries can be directed to the corresponding authors.

## Ethics statement

The study was approved by the first affiliated hospital of anhui medical university Ethics Committee and all subjects gave written informed consent prior to participating in the study. The studies were conducted in accordance with the local legislation and institutional requirements. Written informed consent for participation in this study was provided by the participants’ legal guardians/next of kin. Written informed consent was obtained from the individual(s), and minor(s)’ legal guardian/next of kin, for the publication of any potentially identifiable images or data included in this article.

## Author contributions

HS: Conceptualization, Data curation, Writing – review & editing. HX: Data curation, Formal analysis, Methodology, Writing – original draft. ZP: Data curation, Formal analysis, Methodology, Writing – original draft. YL: Data curation, Formal analysis, Methodology, Writing – original draft. WD: Data curation, Formal analysis, Writing – original draft. RZ: Data curation, Formal analysis, Methodology, Writing – original draft. YS: Data curation, Formal analysis, Methodology, Writing – original draft. ZW: Data curation, Formal analysis, Writing – original draft. JY: Data curation, Formal Analysis, Writing – original draft. HG: Data curation, Formal analysis, Writing – original draft. KY: Data curation, Formal analysis, Methodology, Writing – original draft. JZ: Writing – review & editing, Data curation, Formal analysis. YY: Writing – review & editing, Conceptualization, Data curation, Formal analysis. XL: Writing – original draft, Writing – review & editing, Conceptualization, Data curation, Formal analysis.
